# Molecular profiling of chordoma

**DOI:** 10.3892/ijo.2014.2268

**Published:** 2014-01-21

**Authors:** STEFANIE SCHEIL-BERTRAM, ROLAND KAPPLER, ALEXANDRA VON BAER, ERICH HARTWIG, MICHAEL SARKAR, MASSIMO SERRA, SILKE BRÜDERLEIN, BETTINA WESTHOFF, INGO MELZNER, BIRGIT BASSALY, JOCHEN HERMS, HEINZ-HERMANN HUGO, MICHAEL SCHULTE, PETER MÖLLER

**Affiliations:** 1 Institute of Pathology, University Hospitals of Ulm;; 2 Institute of Pathology and Cytology, Dr. Horst Schmidt Clinic, Academic Teaching Hospital of University of Mainz, Wiesbaden;; 3 Department of Pediatric Surgery, Dr. von Hauner Children’s Hospital, Ludwig-Maximilian University of Munich, Munich;; 4 Department of Orthopedic Trauma, Hand and Reconstructive Surgery, University Hospitals of Ulm;; 5 Department of Trauma, Hand and Reconstructive Surgery, Ev. Diakonissenanstalt, Karlsruhe;; 6 Department of Trauma and Reconstructive Surgery, Karl-Olga-Krankenhaus, Stuttgart, Germany;; 7 Laboratory of Experimental Oncology, Orthopedic Rizzoli Institute, Bologna, Italy;; 8 Department of Orthopedics, University of Düsseldorf;; 9 Institute of Pathology, University of Giessen;; 10 Department of Translational Brain Research, DZNE (German Center for Neurodegenerative Diseases) and Ludwig-Maximilian University of Munich, Munich;; 11 Department of Neurosurgery, University of Kiel, Kiel;; 12 Department of Trauma and Orthopedic Surgery, Diakoniekrankenhaus, Rotenburg (Wümme), Germany

**Keywords:** chordoma, chondrosarcoma, GeneChip, comparative genomic hybridization, fluorescence *in situ* hybridization

## Abstract

The molecular basis of chordoma is still poorly understood, particularly with respect to differentially expressed genes involved in the primary origin of chordoma. In this study, therefore, we compared the transcriptional expression profile of one sacral chordoma recurrence, two chordoma cell lines (U-CH1 and U-CH2) and one chondrosarcoma cell line (U-CS2) with vertebral disc using a high-density oligonucleotide array. The expression of 65 genes whose mRNA levels differed significantly (p<0.001; ≥6-fold change) between chordoma and control (vertebral disc) was identified. Genes with increased expression in chordoma compared to control and chondrosarcoma were most frequently located on chromosomes 2 (11%), 5 (8%), 1 and 7 (each 6%), whereas interphase cytogenetics of 33 chordomas demonstrated gains of chromosomal material most prevalent on 7q (42%), 12q (21%), 17q (21%), 20q (27%) and 22q (21%). The microarray data were confirmed for selected genes by quantitative polymerase chain reaction analysis. As in other studies, we showed the expression of brachyury. We demonstrate the expression of new potential candidates for chordoma tumorigenesis, such as CD24, ECRG4, RARRES2, IGFBP2, RAP1, HAI2, RAB38, osteopontin, GalNAc-T3, VAMP8 and others. Thus, we identified and validated a set of interesting candidate genes whose differential expression likely plays a role in chordoma.

## Introduction

Chordoma is a rare, low-malignant bone tumor. This unique bone tumor has both epithelial and mesenchymal characteristics (1). Chordomas arise along the spine with hot spots at the upper (skull base 20–30%) and lower (sacro-coccygeal 50–60%) end, and are therefore thought to originate from remnants of the notochord (2). Chordomas grow slowly. However, due to their location, it is difficult to obtain wide-margin resection. Frequently, these tumors recur after surgical treatment. Systemic treatments are largely ineffective and new therapeutic approaches are therefore needed. To date, no targeted therapeutic strategies have been established for chordomas. Recently, however, a phase II study showed a modest antitumor activity of lapatinib in chordoma (3–6).

Chordoma characteristically occurs in adolescence and is rarely found in children. Conventional and molecular cytogenetic analyses revealed chromosomal gains of 7q and losses of 1p and 3p to be the most prominent alterations in chordoma (7). In addition, loss of heterozygosity (LOH) and genome-wide linkage studies have already been successfully used to narrow down and define candidate regions for chordoma development on 1p36.13 and 7q33 (8,9). Some studies focused on gene expression analysis in chordoma. Brachyury (T) was one of these candidates (reviewed in ref. 10), which was knocked down in U-CH1, resulting in striking morphological changes in the tumor cells (11). However, many specific genes or altered transcripts have yet to be determined.

This study comprises a genome-wide cytogenetic analysis of 33 chordomas using comparative genomic hybridization (CGH) and, in selected cases, additional transcript profiling by microarray analysis. We linked these with RT-PCR, immunohistochemistry and FACS analysis. We performed this comprehensive study to determine those genes most differentially expressed in chordoma and thus to establish which had the most promise for translation into clinically useful targets.

## Materials and methods

### Samples

We examined 33 paraffin-embedded chordoma tumor samples (for 7 of which snap-frozen tissue samples were also available) obtained from 26 patients (8 male, 18 female; median age at diagnosis: 66 years), 6 fresh-frozen, conventional chondrosarcomas (6 patients; 4 male, 2 female; median age at diagnosis: 54 years; 1 clivus, 3 femur, 2 pelvis; 3 grade 1, 3 grade 2) and pooled material of short-term cultures of 2 vertebral discs (both male; age 47 and 63 years) from the files of the Institute of Pathology, University Hospitals of Ulm, Germany, Department of Orthopedics, University of Düsseldorf, Germany, Department of Neuropathology, Ludwig-Maximilian University of Munich, Munich, Germany, and Department of Neurosurgery, University of Kiel, Kiel, Germany (Table I).

The chordoma cell lines U-CH1 and U-CH2 were established from sacral chordoma recurrences as described previously (7,12). The chondrosarcoma cell line U-CS2 was established from a chondrosarcoma of the distal femur in a 48-year-old female patient, operated in 2002. One and two years after primary diagnosis, the patient underwent surgery following pulmonary metastasis of the primary grade 2 chondrosarcoma.

### Immunohistochemistry and fluorescence-activated cell sorter analysis (FACS)

Immunostaining was performed using a routine indirect peroxidase method. The following antibodies were applied: TP53 (Dako, Denmark), Ki-67 (Dako), and CD24 (clone 24C02, Dianova, Hamburg, Germany). These antibodies were used at a final concentration of 1–2 *μ*g/ml. For immunohistochemical detection of osteopontin and osteonectin, deparaffinized and ethanol-dehydrated tissue sections were incubated overnight with polyclonal rabbit antibodies to osteonectin (dilution 1:1,000) and osteopontin (dilution 1:3,500) at room temperature. The antibodies were kindly provided by L.W. Fisher, NIH, USA (13). Sections were then incubated with a monoclonal mouse-anti-rabbit antibody (Dako, Glostrup, Denmark) for 30 min followed by signal detection using the Dako ChemMate APAAP system and the Dako TechMate^™^ 500 plus automatic stainer.

FACS analysis was performed according to protocols described previously (14). The following antibodies were applied: CD24 (clone 24C02), CD20 (clone L26, Dako), EMA (clone E29, Dako) and rabbit anti-mouse immunoglobulins (code no. F0313, Dako).

### Cell culture and chromosome preparation

We performed a short-term culture of vertebral discs. The primary cells were seeded, cultured and subcultivated as previously described (7). Metaphase chromosome spreads were prepared from the cell lines and from primary blood cell cultures of healthy donors (for CGH experiments) using standard protocols (7). Cells were karyotyped using conventional GTG-banding techniques according to the 1995 ISCN nomenclature.

### Comparative genomic hybridization (CGH) and fluorescence in situ hybridization (FISH)

All seven chordoma samples were available as paraffin-embedded tissue. In addition, seven tissue samples were available as fresh-frozen samples. Histological evaluation of these samples revealed an estimated tumor cell content of ≥90%. CGH analysis was carried out according to the protocol previously described in detail (7). Image acquisition and processing were performed with the image analysis system ISIS (MetaSystems, Altlussheim, Germany).

FISH was performed on imprint cytology slides and 5-*μ*m sections of paraffin-embedded tumor material. The commercially available combined probe m-bcr/abl with assignment to 9q34 (*ABL* locus) and 22q11.2 (*BCR* locus), and the indirect labeled probes assigned to loci 7cen, 1p36 (all probes by Q-Biogene, Illkirch Cedex, France), and the Her2/*neu* probe (Zytomed, Germany) were applied. Additionally, we used the following YAC clones obtained from the CEPH YAC library: 801_A_8 (3p14.2), 724_G_5 (*RHEB*, 7q36), 798_G_8 (8p12), 751_A_4 (*MDM2*, 12q14.3–q15), 984_D_2 (12q22–q24), 763_A_3 (22q12), and 949_A_7 (Xp11.4) (7,15). FISH experiments were performed as dual-color hybridization as previously described (7).

### RNA preparation and gene expression analyses

Fresh-frozen tissue and cell culture samples were homogenized and total RNA was isolated using the RNeasy Mini kit (Qiagen, Valencia, CA, USA) according to the manufacturer’s instructions. Total RNA was quantitated by ultraviolet absorbance at 260 and 280 nm and its integrity was assessed by means of agarose gel electrophoresis.

### Oligonucleotide array

Total RNA of two pooled vertebral discs, three chordoma recurrences, and the novel chondrosarcoma cell line U-CS2, grade 2, were subjected to gene expression analysis using the high-density oligonucleotide array U133 set (Affymetrix, Santa Clara, CA, USA), which contains a probe set for ∼33,000 well-substantiated human genes. Equal amounts (5 *μ*g) of total RNA of tumors and control samples were sent to the German Resource Center (RZPD, Berlin, Germany). Labeling of total RNA, testing of synthesized cDNA (IVT Ambion’s T7 Megascript kit, Roche), of labeled probes, signal detection and data acquisition was performed as described (16). The Microarray Analysis Suite (MAS) 5.0 software (Affymetrix) was used to calculate the gene expression levels. The Affymetrix Gene Expression Assay has been shown to identify X-fold changes that are >2 for 98% of the time. Based on the observations, robust changes can be identified by selecting transcripts with a fold change of >2 for increases and <2 for decreases, which correspond to a signal log ratio of 1 and −1, respectively.

### cDNA microarray analysis

Expression analysis of four chordomas (one primary and three recurrences) and three chondrosarcomas (Table I), as well as pooled material of short-term culture of vertebral disc as a reference, was performed using a cDNA microarray containing 1,000 human genes involved in hedgehog signaling and cancer (17). Hybridization experiments and signal detection were performed as described above (17).

Image analysis, spot finding and data acquisition were performed with the ImaGene 4.0 software package (BioDiscovery, Los Angeles, CA, USA). Mean signal intensities for each spot were corrected by subtracting the mean signal of local background. Normalization was performed by equalizing the overall intensities of both dyes. The resulting data were used to calculate the ratio of gene expression in tumors versus vertebral disc.

### Real-time reverse transcription polymerase chain reaction (RT-PCR)

Total RNA of cases 1-3R, 6 and 20, U-CS2, six chondrosarcomas, low-grade and vertebral disc was amplified using the RiboAmp RNA amplification kit (Arcturus, Mountain View, CA, USA) according to the manufacturer’s protocol. The RT-PCR reactions were carried out in a final volume of 20 *μ*l containing 25 ng cDNA, 300–900 pmol of each primer (RARRES2 and KRT18 300/300 nM; T1A and ECRG4 900/900 nM; T, IGFBP2 and CD24 300/300 nM), and 10 *μ*l SYBR green PCR master mix (Applied Biosystems, UK) in a thermocycler (iCycler, Bio-Rad, Germany). For PCR experiments, reverse and forward primers were selected for the following genes: T brachyury (mouse) homolog (T; Gene bank accession no. NM_003181.2), CD24 antigen (CD24; Gene bank accession no. NM_013230.2), insulin-like growth factor binding protein 2 (IGFBP2; Gene bank accession no. NM_000597.2), retinoic acid receptor responder 2 (RARRES2; Gene bank accession no. NM_002889.3), esophageal cancer-related gene 4 protein (ECRG4; Gene bank accession no. NM_032411.2), keratin 18 (KRT18; Gene bank accession no. NM_000224.2), podoplanin/T1A-2 lung type-I cell membrane-associated glycoprotein (PDPN/T1A2; Gene bank accession no. NM_006474.4), and osteopontin (SSP1; Gene bank accession no. NM_001040058.1). For all primer sets, hot start PCR was performed with an initial denaturation step at 95°C for 5 min. This was followed by 40 cycles at 95°C for 20 sec, 60°C for 20 sec and 72°C for 20 sec. Final extension was carried out for 10 min at 72°C. Vertebral disc was used as calibrator.

## Results

### Morphology and cytogenetics of U-CH2

**Figure 1.**
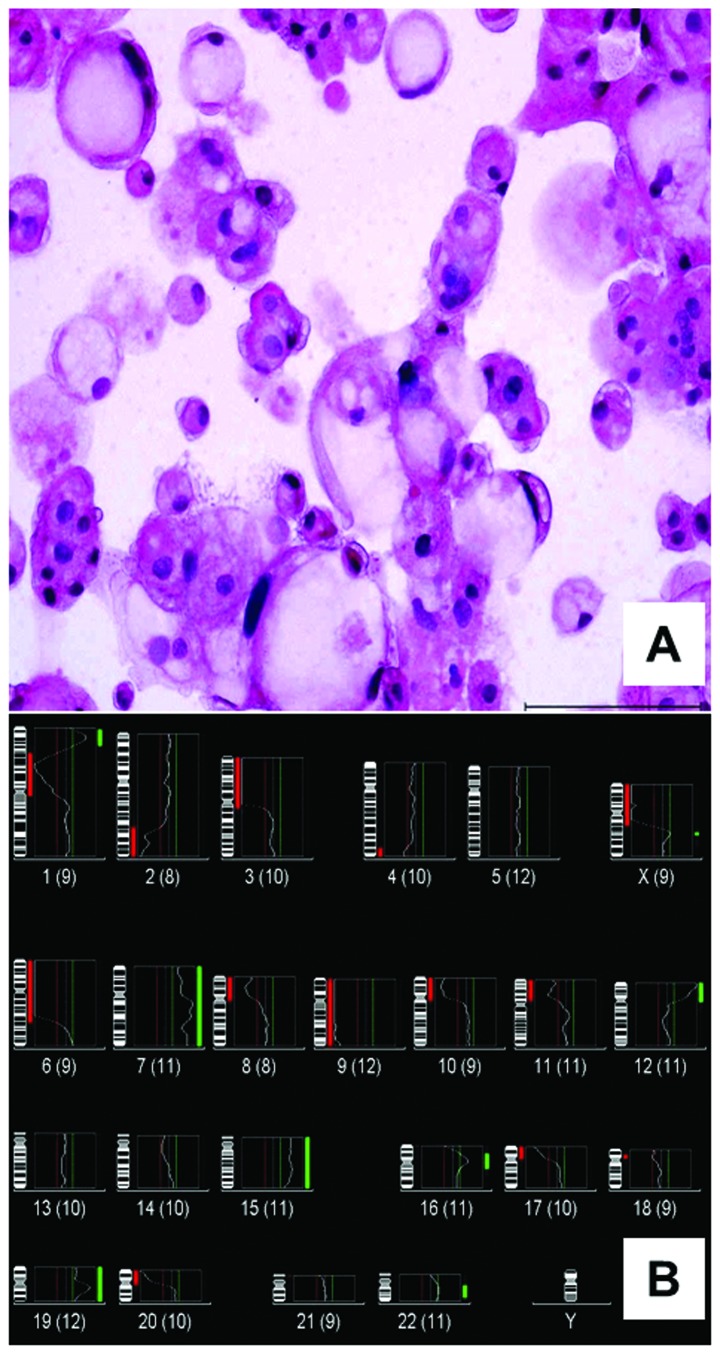
(A) Cytology of the U-CH2 chordoma cell line. (B) CGH analysis of U-CH2. The gains are given in green, the losses in red.

U-CH2 was established from the first recurrence of a chordoma in a 72-year-old woman, whose primary tumor had been operated one year previously (case 3, Table I). Initially, U-CH2 had a doubling time of about four weeks. After 11 passages, the cells maintained a doubling time of approximately one week. Microscopically, U-CH2 is comprised of typical physaliphorous cells (Fig. 1A). CGH of genomic DNA isolated from U-CH2 (cell culture passage no. 2–3) revealed the *rev ish* karyotype enh(1p34.2–p36.1,7,12p,15q,Xq), *dim*(1p11–p31,2q32–q36,3p,4q34–q35,6p-q22,8p,9,10p,11p,17p,20p,Xp) (Fig. 1B). Using M-FISH, 20 out of 38 metaphase spreads (cell culture passage nos. 2–3) could be analyzed demonstrating the following clonal aberrations: t(1;19),t(1;8),del(2)(q),del(4)(p),der(7),t(8;15),t(10;17),der(12)t(8;12),t(7;13), t(14;?),der(16),t(20;22),der(X) t(X;18). Furthermore, we detected non-identified double minutes.

### Molecular cytogenetics in chordomas

**Figure 2.**
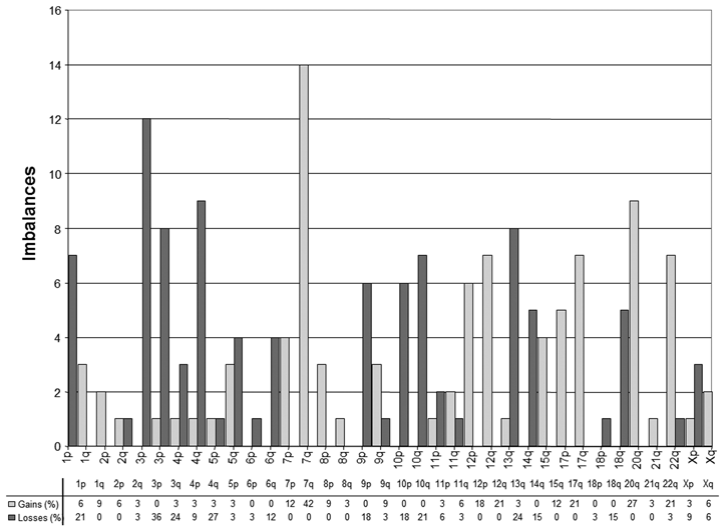
Summary of chromosomal imbalances in our cohort of 33 chordomas.

The CGH findings of 33 chordomas are shown in Table II. Overall, we found 166 chromosomal aberrations (0–14 per tumor; median 4 per tumor) in 33 chordomas. On average, 4.1 losses and 4 gains were detected per tumor. Chromosomal losses occurred most frequently at 1p (21%), 3p (36%), 4q (27%), 10q (21%) and 13q (24%). DNA sequence copy number gains were most prevalent at 7q (42%), 12q (21%), 17q (21%), 20q (27%), and 22q (21%). The distribution of deletions, gains and the total number of aberrations in tumors are shown in Table II and Fig. 2.

FISH was performed on 27 samples. In 6 cases (nos. 4R, 7, 14, 15, 24 and 24R) only limited amounts of material were available and thus FISH was not performed. The FISH loci were selected according to the results of CGH. However, FISH analysis revealed no further chromosomal aberrations and confirmed the CGH data (Table II). Most chordomas were nearly diploid, with four exceptions (nos. 1, 18, 26 and 26R). Those tumors were nearly triploid.

Using dual color FISH analysis, we found a high-level DNA amplification of Her2/*neu* (on average 15 signals per cell) in case 18. All nuclei demonstrated >10 signals per nucleus. Interestingly, this case was an abdominal metastasis of a sacral chordoma 9 years after primary diagnosis. The tumor recurred twice during the following 7 months. We checked four further samples with a gain of chromosome 17 (nos. 9, 16, 17R1 and 17R2), but they did not reveal further amplifications of Her2/*neu*.

### Analysis of gene transcript expression in chordoma

**Figure 3.**
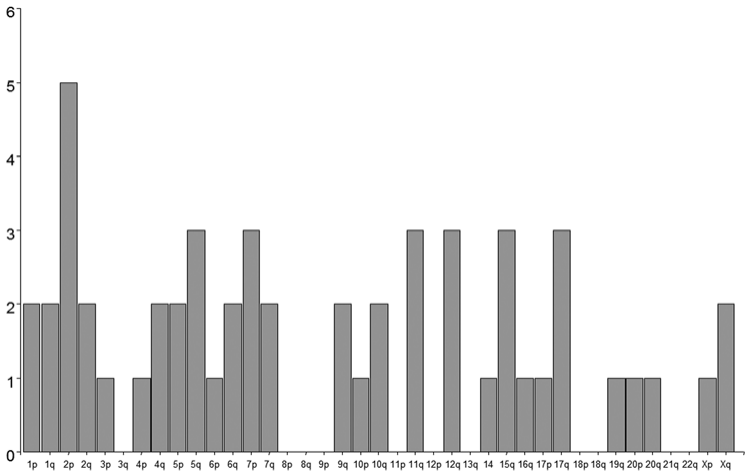
Summary of gene expression analysis of the chromosomal arms of the 65 candidate genes whose mRNA levels differed significantly (p<0.001; ≥6-fold change) between chordoma and control (vertebral disc).

**Figure 4.**
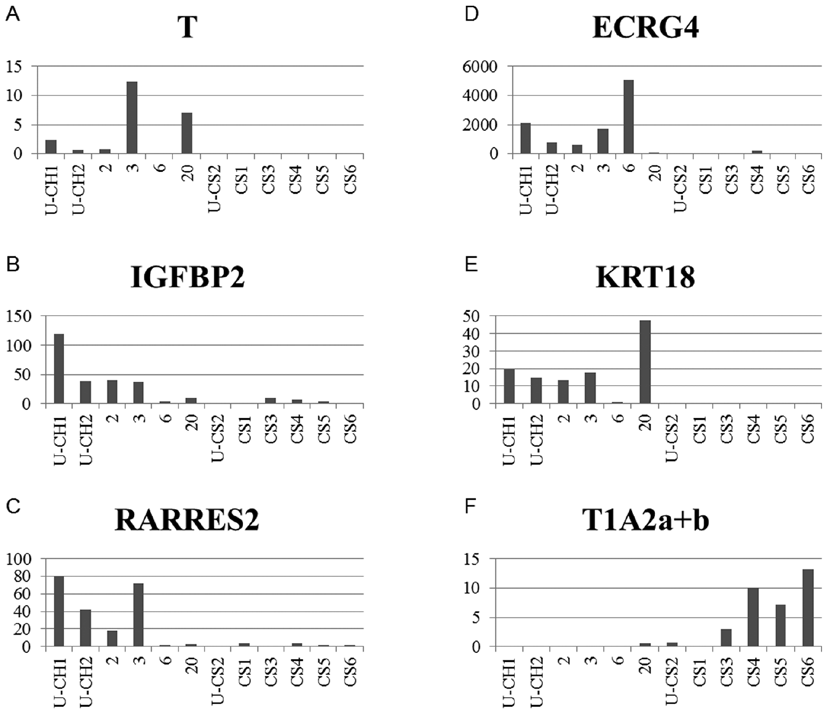
Expression of gene transcripts (RT-RCR) of: (A) T brachyury; (B) IGFBP2; (C) RARRES2; (D) ECRG4; (E) cytokeratin 18, KRT18; and (F) T1A1a–b. Each value is expressed as the ratio of three values of each tumor (6 chordomas of 5 patients; 6 chondrosarcoma of 6 patients)/control (vertebral disc).

**Figure 5.**
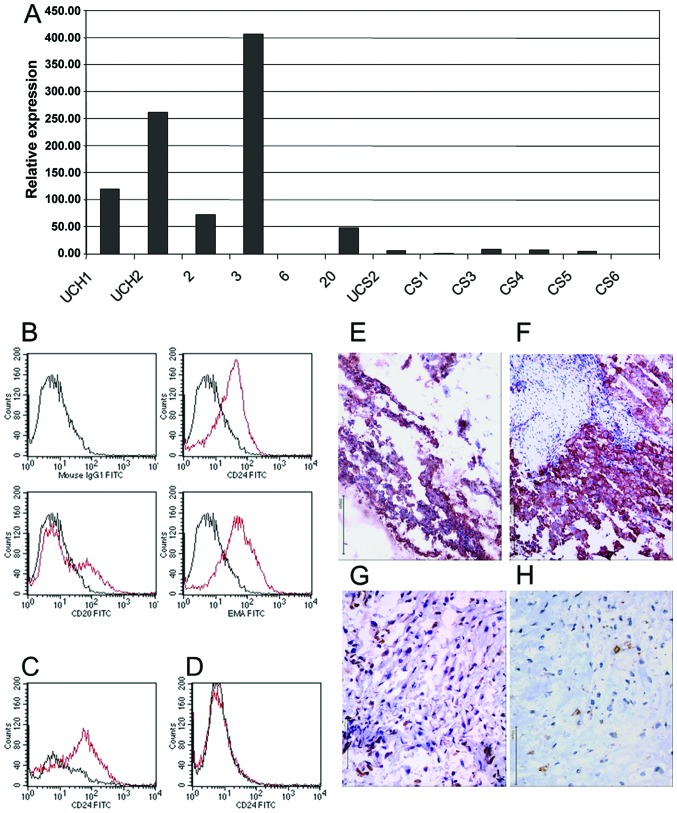
(A) Expression of CD24 transcript (RT-RCR). Each value was expressed as relative expression (n=3) of each tumor (6 chordomas of 5 patients; 10 chondrosarcoma of 10 patients)/control (vertebral disc). FACS analysis of: (B) CD24, CD20, EMA and negative control of the chordoma cell line U-CH2; (C) CD24 analysis of previous published chordoma cell line U-CH1 (7); and (D) the chondrosarcoma cell line U-CS2. Immunohistochemistry of CD24 (clone 24C02) frozen section of: (E) parental tumor of U-CH1 (case 1); (F) chordoma no. 2 (both bars, 200 *μ*m; (G) CS1; and (H) CS6 focally weak immunoreactivity (both bars, 100 *μ*m).

Firstly, we compared the transcriptional profile of ∼33,000 genes in three sacral chordoma recurrences, including the chordoma cell lines (U-CH1 and U-CH2) and the novel chondrosarcoma cell line, U-CS2, with vertebral disc using Affymetrix Human Genome U133 set GeneChips. We identified 65 genes with distinct mRNA levels (p<0.001; ≥6-fold change) of chordoma compared to the control samples (vertebral disc) (Table III and Fig. 3). The genes were most frequently located on chromosome 2 (7/65), 5 (5/65), 1 and 7 (each 4/65) (Fig. 3). The microarray data were corroborated by real-time PCR analysis for selected genes, including six genes [T brachyury (mouse) homolog (T), CD24 antigen (CD24), insulin-like growth factor binding protein 2 (IGFBP2), retinoic acid receptor responder 2 (RARRES2), esophageal cancer-related gene 4 protein (ECRG4) and keratin 18 (KRT18)] with increased expression and one gene (T1A-2 lung type-I cell membrane-associated glycoprotein T1A2) with reduced expression compared to control and chondrosarcoma. The RT-PCR data were determined in an independent series of six chordomas and six chondrosarcomas, including the U-CS2 cell line (Figs. 4 and 5A). These analyses confirmed that the transcript levels of the selected genes which differed significantly between chordoma and chondrosarcoma. One interesting candidate gene in chordoma-genesis is CD24. Using the Affymetrix Human Genome U133 set GeneChip set, CD24 was highly expressed in chordoma (signal: 1409) compared to vertebral disc (p<0.00024; signal: 10) or to U-CS2 (signal: 62.9). With respect to immunohistochemistry and FACS analysis, it was demonstrated that CD24 antigen is highly abundant in all chordomas (Fig. 5B–F), but is absent in conventional skeletal chondrosarcomas. CS6 demonstrated focally weak unspecific background immunoreactivity and we therefore diagnosed a negative CD24 immunoreactivity (Fig. 5H).

Since it has been suggested that sonic hedgehog (SHH) may be involved in chordomagenesis (7), we screened 4 sacral chordomas and 3 chondrosarcomas for differentially expressed genes using a medium-dense cDNA microarray, which comprises genes associated with hedgehog signaling and cancer (17). However, we detected no increase in the expression of SHH and known downstream targets of the hedgehog signaling cascade, such as PTCH1, GLI1, GLI3, D-type cyclins (18), FOXF1 and GADD45a (19). Interestingly, the gene coding for osteopontin (SSP), which has been shown to be transcriptionally activated by GLI1, was upregulated in four out of six chordomas and in one out of three chondrosarcomas in the cDNA microarray analysis (data not shown). Importantly, we demonstrated SSP protein in all chordomas (Table I), but not in chondrosarcomas (n=6) using immunohistochemistry. However, we could not show any prognostic impact of SSP or osteonectin expression in these tumors (data not shown).

## Discussion

In order to identify new candidate genes in chordomagenesis, we performed a combined study of genome-wide analysis of 33 chordomas using CGH and a transcript profile analysis of a subgroup of 6 chordomas compared to 10 chondrosarcomas, grade 1–2. Molecular cytogenetics showed that gains of chromosomal material in chordoma were most prevalent at 7q (42%), 12q (21%), 17q (21%), 20q (27%) and 22q (21%) (Fig. 2). DNA sequence losses occurred most frequently at 1p (21%), 3p (36%), 4q (27%), 10q (21%) and 13q (24%) (Fig. 2). A recent study summarized recurrent cytogenetic copy number alterations published by these different groups (4,7,22,23). Including our data, a consistent recurrent gain of all 101 chordomas studied in these four different cohorts was found on 7q (25–69%), whereas consistent losses were found on 3p (36–75%), 10q (21–65%) and 13q (24–61%). In summary, these tumors are characterized by non-random genomic copy number alterations, where losses are more frequent than gains.

Whereas the CGH analysis demonstrated gains of chromosomal material in chordoma most prevalent at 7q, 12q, 17q, 20q and 22q, the gene transcripts with increased expression compared to control and chondrosarcoma were most frequently located on 2 (11%), 5 (8%), 1 and 7 (each 6%) (Fig. 3). In an earlier study (7), we suggested that oncogenes located on 7q36 might be involved in chordomagenesis. Using GeneChip experiments we could not identify any known oncogene located on 7q36 that is misregulated in chordoma. Furthermore, we could not demonstrate that our former candidate genes, HLXB9 and SHH (7), are overexpressed in chordoma. None of the genes involved in the SHH pathway was transcriptionally activated in chordoma or chondrosarcoma. However, the gene coding for osteopontin (SSP), which has been shown to be transcriptionally activated by GLI1, was upregulated in four chordomas and one chondrosarcoma. SSP has been recognized to be important in the processes of tumorigenicity and metastasis of various cancers (22). Using immunohistochemistry in 24 chordomas obtained from 19 patients, we could not demonstrate any prognostic relevance of SSP expression and prognosis (data not shown).

Another candidate gene found in our GeneChip expression analysis was the transcription factor T brachyury (T), which was highly increased in chordoma compared to vertebral disc or U-CS2 (Table III). This transcription factor is located on 6q27. It influences the cell cycle in different ways to other transcription factors, growth factors, cytokines and kinases and it influences the cell differentiation (10). In several studies, T was identified in chordoma (reviewed in ref. 10). T seems to be the key transcription factor in chordomas. In a very early review, the mechanisms of repair of bone and cartilage were described (23). They summarized that the control of chondrocytic differentiation is affected by the interplay of T, BMP-4, and TGFβ3. T protein is vital for the formation and differentiation of posterior mesoderm and for axial development in all vertebrates (24). The authors demonstrated that T mutant mice or zebrafish die due to, for example, abnormality or lack of the notochord. They found that human T expression was very similar to that found for T in other vertebrate species and was confined to cells derived from the notochord. Chordoma originates from notochordal remnants. A genetic and functional-based study, demonstrated the role of T in the pathogenesis of sporadic chordoma (11). The group summarized that gain of the T locus is common in sporadic chordomas and that expression of this gene is critical for proliferation of chordoma cells *in vitro*. A common single-nucleotide variant in this gene is strongly associated with development of the disease (25). Furthermore, *in vitro* silencing of T induces growth arrest of chordoma cells (25). The authors showed that specific target genes of the transcription factor have been identified through shRNA-mediated silencing followed by global gene expression microarray analyses.

Recently, duplication of the transcription factor T was shown to be associated with the development of chordoma in a few families (26). At any rate, screening for mutations in T (all coding exons and promoter) failed to show any genetic alterations in 23 chordomas (5). Furthermore, amplification of T was described in a subgroup of sporadic chordoma. In line with this, we found that T is overexpressed in chordoma.

Specific target genes of the transcription factor T have been identified through shRNA-mediated silencing followed by global gene expression microarray analyses of 18 chordomas and the cell line U-CH1 (27). These genes include growth factors such as TGFA, FGF1 or EGF. To date, there has been little experience in chordoma with targeting therapy strategies (28) using tyrosine kinase inhibition. In their case report, the authors summarized a total of 4 cases and described a duration of response between 4 and 12 months (28).

Another gene expressed in chordoma and carcinoma is CD24. Two studies focused on aspects of CD24 (small cell lung carcinoma cluster 4 antigen) as a prognostic marker in epithelial malignomas (29,30). In invasive breast cancer, the authors found CD24 expression in 84.6% of cases. In univariate survival analysis, a significant association of CD24 expression with shortened patient overall survival (5-year survival rate 91.9 versus 83.8%; p=0.031; log rank test) and disease-free survival (5-year progression rate 88.3 versus 57.0%; p=0.0008) was demonstrated. Kaplan-Meier curves and Cox regression analysis of their prostate cancer study showed that CD24 expression was strongly linked to significantly earlier disease progression (relative risk, 3.2), which was especially pronounced in organ-confined or moderately differentiated primary prostate tumors. In our cohort of chordoma and chondrosarcoma, we demonstrated by immunohistochemistry and, in part, by FACS analysis that our chordoma cell lines and the fresh-frozen chordomas (n=7) express CD24 protein. Importantly, none of the skeletal chondrosarcomas (n=9) or U-CS2 expressed CD24 antigen demonstrated CD24 immunoreactivity. Further investigations are needed in order to study the prognostic relevance of CD24 protein expression in chordoma.

Another new candidate gene is the esophageal cancer-related gene 4 (*ECRG4/C2ORF40*). To date, this gene has been described in epithelial tumors. Chordoma is a unique bone tumor with both epithelial and mesenchymal characteristics (1). We detected a gene expressed in chordomas that was previously found to be a prognostic marker in various carcinomas (reviewed in ref. 31). In 1998, *ECRG4* was identified from normal esophageal epithelium (32). Several years ago, the encoded protein (augurin) was identified (33). We could not analyze the prognostic impact of ECRG4 expression in chordoma. Further studies are needed to address this issue.

Systemic treatments of chordoma are largely ineffective and new therapeutic approaches are therefore needed. Only very recently, survivin expression has been suggested for use as a potential target gene of angiogenesis in sacral chordoma (34). Consequently, our study indicated a series of 65 genes that are differentially expressed in chordoma. Further studies are needed to validate our set of genes in order to define their possible value as new candidate prognostic and therapeutic targets for chordomas.

## Figures and Tables

**Table I.** Summary of selected clinical data, histopathologic characteristics.

Case/sex/age/tumor status	Localization	Follow-up (Month)	P53 LI	Ki-67 PI	CD24 IR	Osteopontin IR	Osteonectin IR	Microarray data	Real-time PCR
^*^1/M/46/R	Sacral	6	3.1	13.4	+++	++	++	A/K	Y
^*^2/F/77/R	Sacral	31 o Met	3.3	5.5	+++	ND	ND	A/K	Y
^*^3/F/70/P	Sacral	30	3.8	14.4	+++	+	++	K	Y
^*^3R/F/71/R	Sacral	6	5.3	14.2	+	+	++	ND	Y
3R/U-CH2	Sacral		ND	ND	++	ND	ND	A/K	Y
^*^4/F/69/P	Sacral	22 p Mets	ND	ND	ND	ND	ND	ND	ND
^*^4R/F/70/R	Sacral	144	8.3	4.5	ND	++	+	ND	ND
^*^5/F/46/P	Sacral	72	4	5.5	ND	+	++	ND	ND
^*^5R/F/52/R	Sacral	12	2.3	2.1	ND	+	++	ND	ND
^*^6/F/74/R	Sacral	45 DOD	8.3	4.5	++	+	+++	ND	Y
7/M/68/P	Sacral	47	1.7	4.9	ND	+	+++	ND	ND
8/F/60/P	Sacral	8 DOD	6.3	2.6	ND	+	++	ND	ND
9/F/78/P	Sacral	77 DOD	1.9	1.6	ND	ND	ND	ND	ND
10/F/56/R	Sacral	62 DOD	10	4.6	ND	+	+	ND	ND
11/F/65/P	Sacral	92 DOD	2.4	5	ND	ND	ND	ND	ND
12/M/70/P	Sacral	58 R	4.4	2.6	ND	+++	++	ND	ND
13/F/66/R	Sacral	58 DOD	7.9	3.3	ND	ND	ND	ND	ND
14/F/66/R	Sacral	2M DOD	ND	7	ND	++	-	ND	ND
15//M/70/R	Sacral	38	2.4	2.8	ND	+	ND	ND	ND
16/F/63/P	Sacral	23	ND	6.1	ND	+	-	ND	ND
17/F/72/P	Sacral	23	2.1	2.3	ND	+	++	ND	ND
17R1/F/73/R	Sacral/vaginal		4.4	31.5	ND	+++	+++	ND	ND
17R2/F73/Met	Abdominal/perianal		2.4	8.6	ND	++	ND	ND	ND
18/M/65/R/Met	Abdominal/sacral	12	18.8	5.5	ND	++	+++	ND	ND
19/F/17/P	Spinal	36 DOD	5.3	2.7	ND	++	-	ND	ND
20/M/78/P	Spinal	36 R	15	2.1	ND	ND	ND	ND	ND
21/F/63/R	Clivus	13	ND	ND	+++	ND	ND	ND	Y
22/M/52/R	Clivus	52	5.7	10.7	ND	+	++	ND	ND
23/F/57/P	Clivus	26	ND	2	ND	+	+	ND	ND
24/F/67/P	Clivus	12	2.7	2.8	ND	+	+	ND	ND
24R/F/68/R	Clivus		31.3	12.3	ND	+	++	ND	ND
^*^25/M/37/P	Clivus	66	3.4	5.7	ND	ND	ND	ND	ND
^*^26/F/58/P	Clivus	108	14.2	3.2	ND	ND	ND	ND	ND
26R/F/67/R	Clivus		3.3	9.3	ND	++	+	ND	ND

M, male; F, female; P, primary chordoma; R, recurrence and radiotherapy before surgery; Met, metastasis; oMet, bone metastasis; pMet, pulmonary metastasis; DOD, died of disease; CD24 IR, immunoreactivity (figure CD24); PI, proliferation index; ND, no data; A, Affymetrix U133A/B; K, Kappler *et al* (17); Y, yes;

* CGH, FISH and Ki67-PI data published before Scheil *et al* (7).

**Table II.** Summary of CGH and FISH results.

Case	FISH (Mean of FISH-signals/nucleus)		Chromosomal imbalances using CGH	

			Gains	Losses
^*^1	1p36:1.8;	7qter: 4.7;	**7; 8p; 9q34**; 12q24; 15q; 17, 20q	**1p21-p34; 3; 10**; 11; 14q; 18; **22**
	3p14: 1.9;	8p12: 3.8;		
	6cen: 2.7;	9cen: 2.7;		
	7cen: 4.7;	9q34: 3.6;		
	7q34-35: 4.2;	10cen: 1.4;		
	7q36: 4.7;	22q11: 2.0;		
^*^2	7cen: NA;	7qter: 2.7	**7q36**; 20	1p22-p31.3; 3p12-p21; 13q21-q32; 18q22-q23
	7q34-q35: 2.5;			
^*^3	1p36: 3.1;	7qter: 2.9;	**1p34.2-p36; 7p21-qter**; 12p; 15q; **22q**	1p21-p31; 3p; **6q11-q21**; 9p; Xp
	6cen: 1.6;	9q34: 1.9;		
	7cen: 2.8;	22q11: 2.8		
^*^3R	1p36: 3.4;	7qTIM: 3.8;	amp1p34.2-p36; **7**; 12p; 15q; 22q	1p21-p31; 2q33-q36; 3p; 6q11-q21; 9p-q31; Xp
	7cen: 3.2;	7q36: 3.9		
	7q34-q35: 3.9;			
^*^4	1p22: 2.1;	7q34-q35: 3.0;	**7q22-qter**; 12p	-
	7cen: NA;	7q36: 3.3		
	7qTIM: 2.5;			
^*^4R	ND		7q22-qter	3; 4; 5; 9p; 10
^*^5	7cen: NA;	7q36: 2.9	5q23-qter; **7**; 12q24; 20	3; 4q35
	7q34-q35: 2.5;			
^*^5R	7cen: NA;	9q34: 2.2;	5q31-qter; **7q34-qter**; 12q24; 20; **22q**;	-
	7q34-q35: 2.5;	22q11: 2.7	Xq23-qter	
	7q36: 2.4;			
^*^6	1p22: 2.2;	7q36: 4.1;	1q; **3p**; 4q12-q27; 5q; **7; 8pter-q21.1**; 8q24;	-
	3p14: 4.2;	8p12: 4.1;	9q22-qter; 11pter-q22; 12; 13q22-qter; 15q;	
	7cen: NA;	10cen: 2.3;	17q; 21; **22**	
	7q34-q35: 4;	22q12: 4.3		
	7qTIM: 4.5;			
7	ND		5q35; 7q36; 8q24; 9q34; 10q26; 11q25; 12q24; 20q; 22q12-qter; X	4q24-q26; 5q15-q21; 6q11-q15; 13q21
			12q24; 20q;22q12-qter; X	
8	1p36: 1.6;	7qter: 2.8;	-	**1p13-p34; 3p11-p22; 10**; 18q22-qter
	3p14.2: 1.2;	8p22: 2.1;		
	7q36: 2.2;	10cen: 1.5		
9	1p36: 2.7;	8p22: 2.4;	**17**;20q	**3p11-p23**; 3q25-q26; 4q26-q28; 10p15; 10q11-q24
	3p14.2: 1.4;	9q24:1.9;		
	7q36: 2.2;	17qHer2neu: 2.5;		
	7qter: 2.5;	22q11: 2.2		
10	7qter: 2.6;		-	-
	8p22: 2.2			
11	7q36: 2.3;	8p22:: 2.2	-	-
	7qter: 2.4;			
12	7q36: 2;	8p22: 2.1	-	-
	7qter: 2.8;			
13	3p14.2: 1.8;	12qMDM2: 2.1;	-	4q32-qter; 13q14-q21
	7q36: 2.3;	12q22-q24: 2		
	8p22: 2.1;			
14	ND	ND	-	-
15	ND	ND	-	-
16	1p36: 2;	9q34: 2;	**7q34-q36; 17**; 22q12-q13.1	-
	3p14.2: 2.2;	17qHer2neu: 2.8;		
	7q36: 2.9;	22q11: 2.1		
	8p22: 2;			
17	7qter: 2.8;	Xp21.1: 2;	-	-
	8p22: 2;	Xp11.4: 2.2		
17R1	3p14.2: 1.2;	17qHer2neu: 2.8	2p24;2q37; **17p12-qter**; 22q	1p22-p31; **3**; 4p13-p15; 5q13-q21; 9p13-pter;
	8p22: 2.1;			10q21-qter; 11p11.2-p15.3; 14q12-q21; 18q
17R2	3p14.2: 1.8;	9q34: 2;	**17**; 20q11-q13.2	18q23
	7q36: 2.2;	17qHer2neu: 2.7;		
	8p22: 2.1;	22q11: 2.2		
18	3p14.2: 2;	9q34: 3;	1q11-q32; 9q34; **17q**; 20q; 22q	3p14.1-3q25; 4q11-q28; 6q14-q24; 13q; 14q
	7q36:2.4;	17qHer2neu: 15;		
	8p22:2.6;	22q11: 4.4		
19	7q36:2.2;	12qMDM2: 2.1;	4p16	-
	7qter:2.3;	12q22-q24:2		
	8p22: 2.1;			
20	7q36: 2,2;		-	-
	8p22:2.3			
21	7q36: 2.7;	8p22: 2	**7q36**	-
	7qter: 3.2;			
22	3p14.2: 2;	12qMDM2: 2.8;	7q34-q36; 12p13; **12q13-q14;12q22-q24**; 20q12-qter	6p23; 6q31; 9p
	7qter: 2.8;	12q22-q24: 3.9;		
	8p22: 1.9;	Xp21.1: 1.1		
23	7q33: 2.1;	9q34: 2.1;	1q11-q24; 1q32-qter; 5p15; **7q35-q36**; 8p; 12p	3; 10; 14q
	7q36: 2.6;	12q22-q24: 2.3;		
	8q24: 2.7;	22q11: 2.2		
24	ND	ND	-	4q21; 13q21
24R	ND	ND	4q11-q32; 13q21-q22	7q36;12q23-q24; 20q
^*^25	1p22: 2.4;	7q36: 2.4;	12q24	13q21-q31; Xq25-Xqter
	7cen: NA;	9q34: 1.9;		
	7q34-q35: 2.3;	22q11: 2.1		
^*^26	9q34: 2.7;	22q11: 2.8	1q; 11q24-q25	1p; 3; 4; 9p; 10; 13q; 14q; X
26R	7q36: 2.7;	8p22: 2.5	-	-
	7qter: 2.9;			

R, recurrence; ND, no data; CGH data confirmed by FISH are given in boldface type.

**Table III.** Summary of gene transcript expression analysis.^a^

GeneChip probe no.	Locus	Gene U133A/**B**	Expression level			

			Symbol	Chordoma Signal Mean	Chordoma Signal SEM	Fold change
209469_at	6q27	Guanine nucleotide exchange factor for Rap1	Rap1	1043	350	745.1
205150_s_at		T brachyury (mouse) homolog	T	1794	208	245.7
**223748_at**		**Bicarbonate transporter-related protein**	**BTR1**	**4229**	**2884**	**212.5**
220988_s_at		Ribonuclease, RNase A family, 1	RNASE1	3259	339	148.8
213436_at	6q21	Keratin 19	KRT19	4302	278	131.6
201785_at		CD24 antigen (small cell lung carcinoma cluster 4 antigen)	CD24	1409	480.2	122.6
**223631_s_at**		**HAI-2 related small protein /UG=Hs.145362 immortalization-upregulated protein**	**HAI-2**	**1144**	**926**	**114.4**
210982_s_at		Member RAS oncogene family	RAB38	710	156	109.2
204959_at	4q21-q25	C i: Hs.138671 fms-related tyrosine kinase 1 (vascular endothelial growth factor vascular permeability factor receptor)		136	26	91.0
213492_at		Secreted phosphoprotein 1 (osteopontin, bone sialoprotein I, early T-lymphocyte activation 1)	SSP1	4230	1407	89.0
205433_at		C i: Hs.278611 UDP-N-acetyl-α-D-galactosamine:polypeptide N-acetylgalactosaminyltransferase 3	GalNAc-T3	1315	220	71.4
209875_s_at		mRNA, complete cds, clone:SMAP31-12		1578	636	54.8
209994_s_at	2q33-q34	Vesicle-associated membrane protein 8 (endobrevin)	VAMP8	1031	140	53.7
206637_at		Insulin-like growth factor binding protein 2 (36 kDa)	IGFBP2	1281	654	52.7
207315_at		KIAA0644 gene product	KIAA0644	858	492	51.4
210222_s_at		Phosphatidylinositol-4-phosphate 5-kinase, type I, β	PIP5K1B	199	62	37.6
202546_at		Retinoic acid receptor responder (tazarotene induced) 2	RARRES2	1074	252	28.7
205015_s_at		C i: Hs.12969 hypothetical protein		542	337	20.6
206439_at		Integral membrane protein 2A	ITM2A	181	40	20.4
217414_x_at		C i: Hs.80620 guanine nucleotide exchange factor for Rap1; M-Ras-regulated GEF		265	127	16.5
206254_at		C i: Hs.94795 mRNA; cDNA DKFZp564O222 (from clone DKFZp564O222)		1071	720	15.1
220117_at		Keratin 18	KRT18	3020	859	13.7
**239262_at**	**2q12.2**	**Hs.43125 *Homo sapiens* esophageal cancer related gene 4 protein mRNA, complete cds**	**ECRG4**	**672**	**547**	**13.8**
204220_at		C i: Hs.12969 hypothetical protein		509	285	12.8
212843_at		γ-aminobutyric acid (GABA) A receptor, α2	GABRA2	236	60	12.5
209292_at		Hs.75893 ankyrin 3, node of Ranvier (ankyrin G)	Ankyrin G	296	147	9.7
203485_at		Similar to actin binding LIM protein 1/Hs.158203 actin binding LIM protein 1		237	135	9.7
219411_at		C i: Hs.323079 mRNA; cDNA DKFZp564P116 (from clone DKFZp564P116)		332	114	8.9
221595_at		Transcription factor 8 (represses interleukin 2 expression)	TCF8	144	21	8.6
209765_at		Myosin 5C	MYO5C	290	53	8.3
201690_s_at		Ectodermal-neural cortex (with BTB-like domain)	ENC1	719	123	8.0
210674_s_at		ArgAbl-interacting protein ArgBP2, transcript variant 2	ARGBP2	493	195	7.7
219884_at		Pig10 /UG=Hs.104925 ectodermal-neural cortex (with BTB-like domain)	PIG10	313	40	7.4
201839_s_at		RAB3B, member RAS oncogene family	RAB3B	277	152	6.7
219232_s_at		LRP5 mRNA for lipoprotein receptor related protein 5	LRP5	112	8	6.1
215177_s_at		C i: Hs.169401 apolipoprotein E		202	46	6.0
221651_x_at		C i: DNA sequence from clone RP4-761I2 on chromosome 6 contains 3 part of the gene for enhancer of filamentation (HEF1), ESTs, STSs and CpG islands /UG=Hs.80261 enhancer of filamentation 1 (cas-like docking; Crk-associated substrate related)		402	220	5.7
215930_s_at		Actin binding LIM protein 1 (ABLIM), transcript variant ABLIM-s	ABLIM	598	218	5.6
202768_at		C i: DNA sequence from PAC 696H22 on chromosome Xq21.1-21.2. contains a mouse E25-like gene, a kinesin-like pseudogene and ESTs /UG=Hs.17109 integral membrane protein 2A		393	21	5.2
202581_at		Capping protein (actin filament), gelsolin-like	CAPG	910	166	5.1
213539_at		Hypothetical protein (FLJ20330) /UG=Hs.61485 hypothetical protein		153	43	5.0
210089_s_at		Sialyltransferase 9 (CMP-NeuAc:lactosylceramide α-2,3-sialyltransferase; GM3 synthase)	SIAT9	275	71	4.7
210073_at		8D6 antigen (LOC51293)	LOC51293	284	176	4.3
211654_x_at		C i: KIAA0006 gene, partial cds. /UG=Hs.79307 RacCdc42 guanine exchange factor (GEF) 6		197	30	3.8
204309_at		TP53 target gene 1		129	30	3.5
204844_at		BTG family, member 2 (BTG2)	BTG2	163	62	3.5
206178_at		v-maf musculoaponeurotic fibrosarcoma (avian) oncogene homolog	MAF	217	73	3.4
214453_s_at	6q25-q26	Hypothetical protein FLJ10700 (FLJ10700) /UG=Hs.295909 hypothetical protein FLJ10700		277	62	3.3
204526_s_at		Cytovillin 2/UG=Hs.155191 villin 2 (ezrin)	VIL2	683	103	3.2
210582_s_at		NADH dehydrogenase (ubiquinone) 1 α subcomplex, 7 (14.5 kDa, B14.5a)	NDUFA7	209	30	3.0

a U133A selected genes >100 expression and ≥3-fold expression change sacral chordoma recurrences (nos. 1–3) compared to control/baseline array (vertebral disc) and <3-fold expression change chondrosarcoma cell line/baseline array. U133B data are given in boldface type.

## References

[1] Dorfman HD, Czerniak B (1998). Bone tumors.

[2] Mirra JM, Nelson SD, Della Rocca C, Mertens F, Fletcher CDM, Unni KK, Mertens F (2002). Chordoma. Pathology and Genetics of Tumors of Soft Tissue and Bone World Health Organization Classification of Tumors.

[3] Dewaele B, Maggiani F, Floris G, Ampe M, Vanspauwen V, Wozniak A, Debiec-Rychter M, Sciot R (2011). Frequent activation of EGFR in advanced chordomas. Clin Sarcoma Res.

[4] Diaz RJ, Guduk M, Romagnuolo R, Smith CA, Northcott P, Shih D, Berisha F, Flanagan A, Munoz DG, Cusimano MD, Pamir MN, Rutka JT (2012). High-resolution whole-genome analysis of skull base chordomas implicates FHIT loss in chordoma pathogenesis. Neoplasia.

[5] Shalaby AA, Presneau N, Idowu BD, Thompson L, Briggs TR, Tirabosco R, Diss TC, Flanagan AM (2009). Analysis of the fibroblastic growth factor receptor-RAS/RAF/MEK/ERK-ETS2/ brachyury signalling pathway in chordomas. Mod Pathol.

[6] Stacchiotti S, Tamborini E, Lo Vullo S, Bozzi F, Messina A, Morosi C, Casale A, Crippa F, Conca E, Negri T, Palassini E, Marrari A, Palmerini E, Mariani L, Gronchi A, Pilotti S, Casali PG (2013). Phase II study on lapatinib in advanced EGFR-positive chordoma. Ann Oncol.

[7] Scheil S, Bruederlein S, Liehr T, Starke H, Herms J, Schulte M, Moeller P (2001). Genome wide analysis of 16 chordomas by comparative genomic hybridization and cytogenetics of the first human chordoma cell line, U-CH1. Genes Chromosomes Cancer.

[8] Larizza L, Mortini P, Riva P (2005). Update on the cytogenetics and molecular genetics of chordoma. Hered Cancer Clin Pract.

[9] Kelley MJ, Korczak JF, Sheridan E, Yang X, Goldstein AM, Parry DM (2001). Familial chordoma, a tumor of notochordal remnants, is linked to chromosome 7q33. Am J Hum Genet.

[10] Szuhai K, Hogendoorn PC (2012). ‘The chicken or the egg?’ dilemma strikes back for the controlling mechanism in chordoma. J Pathol.

[11] Presneau N, Shalaby A, Ye H, Pillay N, Halai D, Idowu B, Tirabosco R, Whitwell D, Jacques TS, Kindblom LG, Brüderlein S, Möller P, Leithner A, Liegl B, Amary FM, Athanasou NN, Hogendoorn PC, Mertens F, Szuhai K, Flanagan AM (2011). Role of the transcription factor T (brachyury) in the pathogenesis of sporadic chordoma: a genetic and functional-based study. J Pathol.

[12] Bruederlein S, Sommer JB, Meltzer PS, Li S, Osada T, Ng D, Möller P, Alcorta DA, Kelley MJ (2010). Molecular characterization of putative chordoma cell lines. Sarcoma.

[13] Fisher LW, Hawkins GR, Tuross N, Termine JD (1987). Purification and partial characterization of small proteoglycans I and II, bone sialoproteins I and II, and osteonectin from the mineral compartment of developing human bone. J Biol Chem.

[14] Straeter J, Walczak H, Pukrop T, von Müller L, Hasel C, Kornmann M, Mertens T, Möller P (2002). TRAIL and is receptors in the colonic epithelium: a putative role in the defense of viral infections. Gastroenterology.

[15] Gisselsson D, Pålsson E, Höglund M, Domanski H, Mertens F, Pandis N, Sciot R, Dal Cin P, Bridge JA, Mandahl N (2002). Differentially amplified chromosome 12 sequences in low- and high-grade osteosarcoma. Genes Chromosomes Cancer.

[16] Van Gelder RN, von Zastrow ME, Yool A, Dement WC, Barchas JD, Eberwine JH (1990). Amplified RNA synthesized from limited quantities of heterogeneous cDNA. Proc Natl Acad Sci USA.

[17] Kappler R, Hess I, Schlegel J, Hahn H (2004). Transcriptional up-regulation of Gadd45a in Patched-associated medulloblastoma. Int J Oncol.

[18] Ruiz I, Altaba A, Sánchez P, Dahmane N (2002). Gli and hedgehog in cancer: tumours, embryos and stem cells. Nat Rev Cancer.

[19] Kappler R, Calzada-Wack J, Schnitzbauer U, Koleva M, Herwig A, Piontek G, Graedler F, Adamski J, Heinzmann U, Schlegel J, Hemmerlein B, Quintanilla-Martinez L, Hahn H (2003). Molecular characterization of Patched-associated rhabdomyosarcoma. J Pathol.

[20] Hallor KH, Staaf J, Jönsson G, Heidenblad M, Vult von Steyern F, Bauer HC, Ijszenga M, Hogendoorn PC, Mandahl N, Szuhai K, Mertens F (2008). Frequent deletion of the CDKN2A locus in chordoma: analysis of chromosomal imbalances using array comparative genomic hybridisation. Br J Cancer.

[21] Le LP, Nielsen GP, Rosenberg AE, Thomas D, Batten JM, Deshpande V, Schwab J, Duan Z, Xavier RJ, Hornicek FJ, Iafrate AJ (2011). Recurrent chromosomal copy number alterations in sporadic chordomas. PLoS One.

[22] Rittling SR, Chambers AF (2004). Role of ostopontin in tumour progression. Br J Cancer.

[23] Otto WR, Rao J (2004). Tomorrow’s skeleton staff: mesenchymal stem cells and the repair of bone and cartilage. Cell Prolif.

[24] Edwards YH, Putt W, Lekoape KM, Stott D, Fox M, Hopkinson DA, Sowden J (1996). The human homolog T of the mouse T (Brachyury) gene; gene structure, cDNA sequence, and assignment to chromosome 6q27. Genome Res.

[25] Hsu W, Mohyeldin A, Shah SR, Ap Rhys CM, Johnson LF, Sedora-Roman NI, Kosztowski TA, Awad OA, McCarthy EF, Loeb DM, Wolinsky JP, Gokaslan ZL, Quiñones-Hinojosa A (2011). Generation of chordoma cell line JHC7 and the identification of Brachyury as a novel molecular target. J Neurosurg.

[26] Yang XR, Ng D, Alcorta DA, Liebsch NJ, Sheridan E, Li S, Goldstein AM, Parry DM, Kelley MJ (2009). T (brachyury) gene duplication confers major susceptibility to familial chordoma. Nat Genet.

[27] Nelson AC, Pillay N, Henderson S, Presneau N, Tirabosco R, Halai D, Berisha F, Flicek P, Stemple DL, Stern CD, Wardle FC, Flanagan AM (2012). An integrated functional genomics approach identifies the regulatory network directed by brachyury (T) in chordoma. J Pathol.

[28] Launay SG, Chetaille B, Medina F, Perrot D, Nazarian S, Guiramand J, Moureau-Zabotto L, Bertucci F (2011). Efficacy of epidermal growth factor receptor targeting in advanced chordoma: case report and literature review. BMC Cancer.

[29] Kristiansen G, Winzer KJ, Mayordomo E, Bellach J, Schluns K, Denkert C, Dahl E, Pilarsky C, Altevogt P, Guski H, Dietel M (2004). CD24 expression is a new prognostic marker in breast cancer. Clin Cancer Res.

[30] Kristiansen G, Pilarsky C, Pervan J, Sturzebecher B, Stephan C, Jung K, Loening S, Rosenthal A, Dietel M (2004). CD24 expression is a significant predictor of PSA relapse and poor prognosis in low grade or organ confined prostate cancer. Prostate.

[31] Sabatier R, Finetti P, Adelaide J, Guille A, Borg JP, Chaffanet M, Lane J, Birnbaum D, Bertucci F (2011). Down-regulation of ECRG4, a candidate tumor suppressor gene, in human breast cancer. PLoS One.

[32] Su T, Liu H, Lu S (1998). Cloning and identification of cDNA fragments related to human esophageal cancer. China J Oncol.

[33] Mirabeau O, Perlas E, Severini C, Audero E, Gascuel O, Possenti R, Birney E, Rosenthal N, Gross C (2007). Identification of novel peptide hormones in the human proteome by hidden Markov model screening. Genome Res.

[34] Chen C, Yang HL, Chen KW, Wang GL, Lu J, Yuan Q, Gu YP, Luo ZP (2013). High expression of survivin in sacral chordoma. Med Oncol.

